# A randomised controlled trial of a worry intervention for individuals with persistent persecutory delusions

**DOI:** 10.1016/j.jbtep.2009.09.001

**Published:** 2010-03

**Authors:** Chloe Foster, Helen Startup, Laura Potts, Daniel Freeman

**Affiliations:** a Department of Psychology, P.O. Box 077, Institute of Psychiatry, King's College London, Denmark Hill, London SE5 8AF, UK; b Department of Biostatistics, Institute of Psychiatry, Clinical Trials Unit, King's College London, UK

**Keywords:** Delusions, Paranoia, Schizophrenia, Worry, Anxiety, Cognitive therapy

## Abstract

Recent research has shown that worry is associated with distressing paranoia. Therefore, the aim was to target worry in a therapeutic intervention for individuals with delusions. It was predicted that a worry intervention would reduce levels of worry and paranoia distress. Twenty-four individuals with persistent persecutory delusions and high levels of worry were randomly assigned to receive a four session cognitive-behavioural worry intervention (W-CBT) or treatment as usual (TAU). The worry intervention was specifically designed not to target the content of delusions. In this open-label evaluation, assessments of worry and paranoia were conducted at baseline, at one month (end of treatment) and at two months. The worry intervention achieved a statistically significant reduction in worry which was maintained at two month follow up. A significant reduction in delusional distress was also reported. There was an indication that the worry intervention may also reduce the frequency of paranoid thoughts but this was not statistically significant. In the first trial specifically for persecutory delusions, a brief worry intervention was shown to have benefits. The results support a causal role for worry in paranoid experience.

## 1 Introduction

Developments in the understanding of persecutory delusions have the potential to lead to improvements in treatments. Worry, defined as ‘a chain of thoughts and images, negatively affect-laden and relatively uncontrollable’ (Borkovec, Wilkinson, Folensbee, & Lerman, 1983), is a factor that has recently been implicated in paranoid experience. Clinical levels of worry are present in almost two-thirds of individuals with persecutory delusions and the presence of worry is associated with more distressing delusional experience (Bassett, Sperlinger, & Freeman, 2009; Freeman & Garety, 1999; Morrison & Wells, 2007; Startup, Freeman, & Garety, 2007). A catastrophising worry style predicts the occurrence of non-clinical paranoia and the persistence of persecutory delusions (Freeman et al., 2008; Startup et al., 2007). Emphasis is placed on the importance of worry in a cognitive model of persecutory delusions (Freeman, 2007; Freeman & Freeman, 2008). The intriguing implication is that treatment of worry in individuals with persecutory delusions will also lessen paranoia.

Worry has been successfully targeted in people with generalised anxiety disorder using cognitive-behavioural interventions (see review by Covin, Ouimet, Seeds, & Dozois, 2008). We aimed to examine in a small pilot study whether a brief cognitive-behavioural worry intervention has the potential to be effective at reducing levels of worry and delusional distress in individuals with persecutory delusions and clinically significant levels of worry. The main prediction was that the worry intervention would reduce both worry and paranoia distress compared with treatment as usual. A secondary hypothesis was that the worry intervention would reduce the overall occurrence of delusional thoughts. Strengthening the support for the causal role of worry, it was also predicted that changes in worry would be associated with changes in paranoia.

## 2 Method

### 2.1 Participants

The patients with persecutory delusions were recruited from the South London and Maudsley NHS Foundation Trust. The inclusion criteria were: a current persecutory delusion as defined by Freeman and Garety (2000); the delusion had persisted at least six months; a current clinical diagnosis of schizophrenia, schizoaffective disorder or delusional disorder; a clinically significant level of worry, as indicated by scores of 45 or more on the PSWQ (Behar, Alcaine, Zuellig, & Borkovec, 2003; Startup & Erickson, 2006); and aged between 18 and 65. Criteria for exclusion from the trial were: a primary diagnosis of alcohol or substance dependency; organic syndrome or learning disability; a command of spoken English inadequate for engaging in psychological therapy; judged as unable to give informed consent; and currently engaged in any other individual CBT.

### 2.2 Design

Participants were randomly allocated to one of two conditions: a four session worry reduction intervention and treatment as usual (W-CBT), or treatment as usual (TAU). Participants meeting the entry criteria were randomly allocated to each condition in a 1:1 ratio using randomised permuted blocks varying from two to four (carried out by a researcher independent of the team). Random allocation followed completion of the baseline assessment (a sealed envelope was opened). Data were collected at three time points: at baseline assessment before randomisation, at one month from randomisation and at two months from randomisation. The assessments were carried out by the therapist (CF) and were, therefore, not blind. Ethical approval for the study was obtained from the Joint South London and Maudsley and the Institute of Psychiatry NHS Research Ethics Committee. Data collection was carried out from June 2007 to April 2008.

### 2.3 Treatment and control conditions

Participants in the W-CBT arm of the trial were offered four sessions over one month. The worry reduction strategies included were (i) indicated in the literature to be effective at reducing worry, either alone or in conjunction with other anxiety management strategies; (ii) did not challenge or review the delusion itself; and (iii) had been used by the authors in clinical practice. Key influences were Borkovec, Ray, and Stober (1998), Butler, Gelder, Hibbert, Cullington, and Klimes (1987), Dugas and Ladouceur (1998), Leahy (2006), and Wells (1997). The main techniques were psychoeduction about worry, reviewing of positive and negative beliefs about worry, increasing awareness of the initiation of worry and identification of individual triggers, learning to ‘let go’ of worry, use of worry periods, substituting problem-solving in place of worry, and relaxation exercises. A simple individualised formulation of each person's worry was developed and homework between sessions was agreed. Written information was provided in the form of a leaflet called ‘Winning against Worry’. The therapy was provided by the first author under the supervision of the two other clinical psychologists.

TAU consisted of standard care, delivered according to national and local service protocols and guidelines. During hospitalisation, TAU usually involves prescription of anti-psychotic medication, occupational therapy activities and exercise groups. Following discharge, the level of TAU varies according to the needs of the individual. However, this usually consists of prescription of anti-psychotic medication, visits from a community mental health worker and monthly outpatient appointments with a psychiatrist.

### 2.4 Outcome measures

#### 2.4.1 Penn State Worry Questionnaire (PSWQ; Meyer, Miller, Metzger, & Borkovec, 1990)

The PSWQ is the most established worry questionnaire. It is designed to capture the generality, excessiveness and uncontrollability of worry. Respondents are asked to indicate how typical sixteen statements are of them on a five-point scale, ranging from “not at all typical of me” to “very typical of me”. A high score represents a greater degree of worry. The PSWQ has demonstrated sensitivity to change across both 6-week and 12-week therapeutic interventions for GAD (Borkovec & Costello, 1993).

#### 2.4.2 Psychotic Symptoms Rating Scale: Delusions Subscale (PSYRATS; Haddock, McCarron, Tarrier, & Faragher, 1999)

The PSYRATS, increasingly used in psychosis research (e.g. Lewis et al., 2002), provides a multi-dimensional interviewer rating of delusional beliefs. Each of the six items are rated on a five-point ordinal scale (0–4) and concern the past week. Items on the PSYRATS relating to preoccupation, duration, conviction and disruption load onto Factor 1, labelled ‘cognitive interpretation’, and items relating to amount and intensity of distress load onto Factor 2, labelled ‘emotional impact’. The factor structure was confirmed in a study of over two hundred and fifty individuals presenting with acute first episodes of schizophrenia (Drake, Haddock, Tarrier, Bentall, & Lewis, 2007).

#### 2.4.3 Green et al. Paranoid Thought Scales (GPTS, Green et al., 2008)

The GPTS comprises two 16-item scales: Part A, which assesses social ideas of reference, and Part B, which assesses ideas of persecution. Respondents are asked to indicate on a five-point scale from one (not at all) to five (totally) how often they have experienced each paranoid thought over the past month. Higher scores represent a greater degree of delusional ideation. Within the GPTS are eight-item subscales of conviction, preoccupation, and distress. Convergent validity of the GPTS with the Paranoia Scale (Fenigstein & Vanable, 1992) and the Psychotic Symptoms Rating Scale (Haddock et al., 1999) has been shown.

### 2.5 Intellectual functioning

#### 2.5.1 Wechsler Test of Adult Reading (WTAR; Wechsler, 2001)

The WTAR was used as an assessment of premorbid intellectual functioning and consists of 50 words with irregular pronunciations which the participant is required to read aloud.

### 2.6 Statistical analysis

The outcomes (PSWQ, PSYRATS, GPTS) at one month and two months post-randomisation were modelled by the use of multilevel linear regression. Multilevel linear regression was used as the data produced were longitudinal in nature, and participants were included as a random effect. An advantage of this approach is that it can easily be used if any data are missing and, therefore, provides a way to achieve a fully multilevel analysis of repeated measures with incomplete data (Van Der Leeden, 1998). The models include the respective baseline measurement to control for pre-treatment differences, treatment group (represented by one dummy variable), time (since the one month assessment), and a treatment time interaction term as an explanatory variable. As an interaction term was fitted in all models, the coefficient of the treatment group represented the mean difference between the treatment groups at the one month assessment adjusting for any baseline differences. The models were fitted in Stata (version 10) (Stata Corporation, 2007) using the xtmixed command. In all the analyses, a multilevel model with random intercepts was applied as it was deemed a more appropriate fit than a random coefficient model due to reduced standard errors of within and between patient variance in comparison to the random coefficient model. A full analysis set of participants was used following ICH topic E9. Randomised participants were excluded from the full analyses set if they provided no data post-randomisation. The association between change in worry and in paranoia was examined using Kendalls's tau; this non-parametric measure was used because Kendall's tau is more robust to outliers compared with Pearson's product moment correlation coefficient.

### 2.7 Power analysis

Before the start of the trial, a power analysis was conducted for change in worry based on the planned analysis detailed above. This indicted that a sample size of 24 participants (12 in each arm) would be needed to achieve a clinically important effect size of 0.9, based on a mean difference between interventions of 9 points and a common standard deviation of 10 at one and two months in the primary analysis of the Penn State Worry Questionnaire, at 90% power and 5% (2 sided) significance level. Machin, Campbell, Fayers, and Pinol (1997) suggest that correlations of 0.6–0.75 between baseline and outcome measurement are common. Therefore, a conservative correlation of 0.6 was applied in this sample size calculation. Other assumptions were that there would be no lost to follow up or withdrawals for the duration of the study.

## 3 Results

**Fig. 1:**
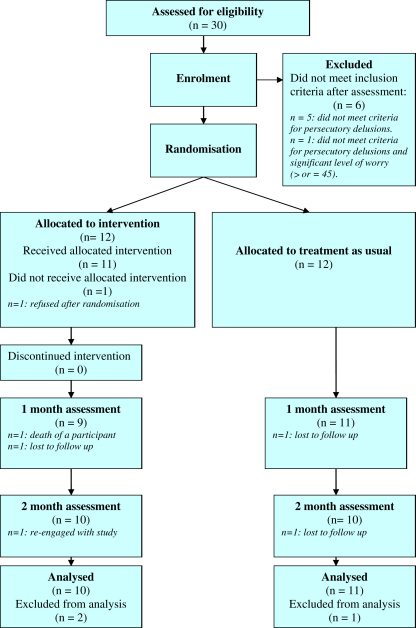
CONSORT flow diagram.

The flow of participants through the trial is shown in Fig. 1.

### 3.1 Demographic and clinical characteristics of the participants

Demographic details are presented in Table 1. The participants were very similar to those in other studies of persistent psychosis; the mean age was approximately forty years old, most were single, and there were high rates of unemployment. The main diagnosis was schizophrenia (*n* = 21, 88%) and the majority of participants were outpatients (*n* = 22, 92%).

### 3.2 Intervention effects: worry

The assessment scores at each time point are displayed in Table 2.

**Fig. 2:**
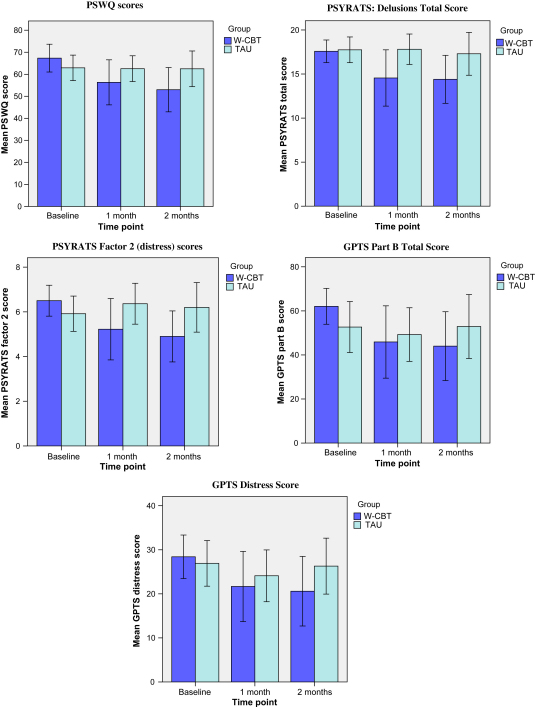
Bar charts showing mean scores (95% CI error bars) for the outcome measures (a) PSWQ scores; (b) PSYRATS: delusions total score; (c) PSYRATS Factor 2 (distress) scores; (d) GPTS Part B Total Score; (e) GPTS distress score.

Within the W-CBT arm of the trial, mean scores on the Penn State Worry Questionnaire reduced by 11.0 points at one month and 14.3 points at two months compared to baseline scores. Within TAU, mean worry score on the PSWQ reduced by 0.3 points at the one month assessment and 0.4 points at the two month assessment. This is illustrated in Fig. 2. The outcome of the PSWQ at one month and two months post-randomisation was modelled by a random intercept multilevel model with patients included as a random effect (see Table 3). The results indicate that W-CBT reduced worry by 10.0 points (95% CI: −18.8, −1.2, *p* = 0.025, SE = 4.48) in comparison to TAU adjusting for baseline differences. The results failed to show a significant change in PSWQ by time and by a treatment time interaction, indicated by non-significant *p*-values. This indicates that W-CBT reduced worry by ten points in comparison to TAU post-treatment and this difference was maintained at two month follow up when accounting for baseline scores.

### 3.3 Intervention effects: paranoia

Within the W-CBT arm of the trial, mean total scores on the PSYRATS reduced by 3.0 points at one month assessment and 3.2 points at two month assessment as compared to baseline scores. Within TAU, there was no change in mean total PSYRATS scores at one month assessment and there was a reduction of 0.5 points at two month assessment compared to baseline scores, as illustrated in Fig. 2. Fitting a random intercept model indicated that W-CBT reduced the PSYRATS total score by 2.9 points (95% CI: −5.3, −0.6, *p* = 0.015, SE = 1.20) points in comparison to TAU when adjusting for baseline differences (see Table 3). The results failed to show a significant change in PSYRATS total score by time and by a treatment time interaction indicated by non-significant *p*-values. Therefore, W-CBT reduced PSYRATS total score by 2.9 points in comparison to TAU post-treatment and this difference was maintained at two month follow up.

Factor 2 (emotional distress) of the PSYRATS offers a summary score of two distress related items; intensity of distress and amount of distress. Within the W-CBT arm of the trial, mean distress scores on the PSYRATS reduced by 1.3 points at one month assessment and 1.6 points at two month assessment compared to baseline scores. Within TAU, there was an increase in mean distress PSYRATS scores by 0.5 points at one month assessment and 0.3 points at two month assessment as compared to baseline scores, as illustrated in Fig. 2. Fitting a random intercept model showed that W-CBT reduced PSYRATS Factor 2 by 1.7 points (95% CI: −2.8, −0.6, *p*-value = 0.003, SE = 0.57) in comparison to treatment as usual when adjusting for baseline differences (see Table 3). The results failed to show a significant change in PSYRATS Factor 2 by time and by a treatment time interaction indicated by non-significant *p*-values. Therefore, W-CBT reduced Factor 2 scores by 1.7 points in comparison to TAU post-treatment and this difference was maintained at two months follow up when accounting for baseline differences.

Within the W-CBT arm of the trial, mean distress subscale scores on the GPTS reduced by 6.7 points at one month assessment and 7.8 points at two month assessment compared to baseline scores. Within TAU, mean distress subscale scores on the GPTS reduced by 2.8 points at one month assessment and 0.6 points at two month assessment compared to baseline scores, as illustrated in Fig. 2. Fitting a random intercept model for the dependent outcome GPTS distress subscale indicated that W-CBT failed to show a significant reduction in distress (adjusted mean difference = −4.6, 95% CI: −13.0, 3.8, *p* = 0.285, SE = 4.28) compared to TAU when adjusting for baseline differences (see Table 3).

Part B of the GPTS is a measure of persecutory ideation. Within the W-CBT arm of the trial, mean Part B scores on the GPTS reduced by 16.2 points at one month assessment and 18.1 points at two month assessment compared to baseline scores. Within TAU, mean Part B scores on the GPTS reduced by 3.4 points at the one month assessment and increased by 0.2 points at the two month assessment compared to scores at baseline assessment, as illustrated in Fig. 2. Fitting a random intercept model for the dependent outcome GPTS Part B indicated that W-CBT failed to show a significant reduction in persecutory ideation (adjusted mean difference = −10.5, 95% CI: −28.6, 7.6, *p*-value = 0.255, SE = 9.23) in comparison to TAU adjusting for baseline differences (see Table 3).

### 3.4 Association of change in worry and paranoia

Associations between changes in worry and changes in persecutory thoughts are reported in Table 4. It can be seen that these associations were positive and that they mostly reached statistical significance; reductions in worry were associated with reductions in paranoia.

### 3.5 Medication

Of the 24 participants in the RCT, 21 were taking anti-psychotic medication; 10 of the individuals in TAU and 11 of those who received W-CBT. Within the TAU group, there were no changes in medication reported during the trial. Within the W-CBT condition, one participant's medication was increased by 5 mg and one participant's medication was increased by 10 mg per day.

### 3.6 Adverse events

No participant had to be withdrawn from the trial. One participant who received W-CBT died following the final therapy session. This death was from an alcohol-related accidental fall that caused a major head injury. Suicidal intent was not suspected and the death was unrelated to the trial.

## 4 Discussion

There is large heterogeneity in the presenting problems of people with psychosis. We have reduced this complexity by focussing on one common experience: persecutory delusions. This is the first report of a randomised controlled trial specifically for persecutory delusions. A key psychological factor identified from the theoretical literature was targeted in a brief therapeutic intervention. A worry intervention was evaluated for its effects on both worry and persecutory delusions.

An important outcome on its own is that a reduction in worry was achieved which was maintained at the short-term follow up. However, the results also indicated that the worry intervention had an impact on paranoid experience. Because of the previous research findings of an association of worry and persecutory delusion distress (Bassett et al., 2009; Freeman & Garety, 1999; Morrison & Wells, 2007; Startup et al., 2007), the analysis focussed upon the distress associated with paranoia as an outcome. The intervention achieved a significant reduction in persecutory delusions, especially the associated distress, as assessed by the PSYRATS. The intervention group also showed reductions on the self-report paranoia questionnaire, while the control group remained stable, although these results did not reach statistical significance. Less change would be expected on the self-report paranoia measure since it concerns the past month (the PSYRATS concerns the past week) and each participant was only in the trial for a total of two months. The intervention shows great promise, particularly given its brevity and the severe experiences reported by the trial patients.

There was a high level of engagement with therapy; all individuals who started treatment attended all four sessions. The advantages of a focus on worry are that it does not challenge the delusion, it targets a problem reported by patients, and by talking about an experience widely recognised as common in the general population it is normalising. It may be an extremely helpful approach before directly addressing the delusional belief. Alternatively, an extended worry intervention, including reviewing, in the manner of a worry, the likelihood of the persecutory fear occurring, may alone have significant effects.

From a theoretical perspective, it is of note that finding an effect of the worry intervention on paranoid experience supports the cognitive model. This was substantiated by finding an association of change in worry with change in paranoia. The plausible direction of the relationship here – because the intervention did not target the delusion – is that changes in worry produced changes in paranoia. The trial demonstrated change in a mechanism, worry, thought to underlie paranoia, which led to change in persecutory delusions.

However, the pilot study suffered from a number of methodological weaknesses. The small sample size lessened the power to detect significant changes in paranoia, which was exacerbated by loss of participants to follow up. It also made it more likely that there were would be baseline differences between the two groups, which was seen in the intervention group initially having higher levels of worry and paranoia. Other important methodological weaknesses were the absence of blinding of the assessments, the use of a single therapist, and no monitoring of therapy adherence or competence. It would also have been interesting to look at longer term outcomes. A larger and more methodologically robust trial is now required.

## Figures and Tables

**Table 1:** The demographic characteristics of the participants.

	W-CBT (*n* = 12)	TAU (*n* = 12)
Age		
Mean age in years	40.0	39.1
Standard deviation	10.0	9.2

Sex (*n*)		
Male	7 (58%)	7 (58%)
Female	5 (42%)	5 (42%)

Ethnicity (*n*)		
White British	4 (33%)	4 (33%)
White other	2 (17%)	0 (0%)
Black British	2 (17%)	5 (42%)
Black African	1 (8%)	3 (25%)
Asian	2 (17%)	0 (0%)
South American	1 (8%)	0 (0%)

Employment (*n*)		
Employed	1 (8%)	1 (8%)
Employed p/t	0 (0%)	1 (8%)
Voluntary employment	1 (8%)	0 (0%)
Unemployed	8 (67%)	9 (75%)
Student	2 (17%)	1 (8%)

Marital status (*n*)		
Single	10 (83%)	11 (92%)
Married	0 (0%)	1 (8%)
Divorced/separated	1 (8%)	0 (0%)
Cohabiting	1 (8%)	0 (0%)

IQ		
Mean	98.4	94.2
Standard deviation	8.5	13.6

**Table 2:** Summary statistics for the assessment measures.

Measure	Time point	W-CBT			TAU		
		*N*	Mean	SD	*N*	Mean	SD
PSWQ							
	Baseline	12	67.3	9.9	12	62.9	9.1
	1 month	9	56.3	13.3	11	62.6	8.7
	2 months	10	53.0	14.1	10	62.5	11.3

PSYRATS							
Total	Baseline	12	17.6	2.0	12	17.8	2.3
	1 month	9	14.6	4.2	11	17.8	2.6
	2 months	10	14.4	3.8	10	17.3	3.4

Factor 1 (frequency/duration)	Baseline	12	11.1	1.7	12	11.8	1.8
	1 month	9	9.3	2.7	11	11.4	1.9
	2 months	10	9.5	2.7	10	11.1	2.4

Factor 2 (distress)	Baseline	12	6.5	1.1	12	5.9	1.2
	1 month	9	5.2	1.8	11	6.4	1.4
	2 months	10	4.9	1.6	10	6.2	1.5

GPTS							
Total	Baseline	12	112.9	23.0	12	100.6	32.7
	1 month	9	86.8	41.4	10	89.7	26.1
	2 months	10	80.0	40.9	10	100.9	31.7

Part A (social reference)	Baseline	12	50.4	15.5	12	47.9	17.1
	1 month	9	40.9	22.3	10	41.0	9.3
	2 months	10	36.0	21.3	10	48.0	13.5

Part B (persecution)	Baseline	12	62.1	12.8	12	52.7	18.2
	1 month	9	45.9	21.4	11	49.3	18.1
	2 months	10	44.0	21.8	10	52.9	20.3

Distress	Baseline	12	28.4	7.8	12	26.9	8.2
	1 month	9	21.7	10.4	10	24.1	8.2
	2 months	10	20.6	11.0	10	26.3	8.9

**Table 3:** Results of the random intercept multilevel models.

	Coefficient	SE	*p*-value	95% CI
Worry (PSWQ)				
PSWQ baseline	0.79	0.22	<0.001	(0.35, 1.23)
CBT worry intervention	−10.00	4.48	0.025	(−18.77, −1.23)
Time	0.79	2.46	0.746	(−4.02, 5.60)
Time by CBT worry intervention	−5.03	3.57	0.159	(−12.03, 1.97)
Constant	14.12	13.99	0.313	(−13.29, 41.53)
Between patient SD	7.90	–	–	–
Within patient SD	5.54	–	–	–
Intraclass correlation	0.67	–	–	–

Delusion (PSYRATS)				
PSYRATS total score baseline	1.02	0.24	<0.001	(0.55, 1.50)
CBT worry intervention	−2.91	1.20	0.015	(−5.27, −0.56)
Time	−0.31	0.86	0.722	(−2.00, 1.38)
Time by CBT worry intervention	0.32	1.26	0.802	(−2.14, 2.78)
Constant	−0.61	4.42	0.891	(−9.27, 8.06)
Between patient SD	1.84	–	–	–
Within patient SD	1.96	–	–	–
Intraclass correlation	0.47	–	–	–

Delusion (PSYRATS – Distress)				
PSYRATS Factor 2 baseline	−0.81	0.20	<0.001	(0.42, 1.20)
CBT worry intervention	−1.66	0.57	0.003	(−2.77, −0.56)
Time	−0.09	0.41	0.826	(−0.90, 0.72)
Time by CBT worry intervention	−0.11	0.60	0.851	(−1.28, 1.06)
Constant	1.53	1.25	0.224	(−0.93, 3.98)
Between patient SD	0.81	–	–	–
Within patient SD	0.93	–	–	–
Intraclass correlation	0.43	–	–	–

Paranoia (GPTS – distress)				
Green Paranoid Thoughts Scale distress score baseline	0.40	0.25	0.107	(−0.09, 0.90)
CBT worry intervention	−4.58	4.28	0.285	(−12.97. 3.82)
Time	2.20	1.76	0.212	(−1.26, 5.66)
Time by CBT worry intervention	−2.01	2.56	0.432	(−7.03, 3.00)
Constant	13.3	7.31	0.068	(−0.99, 27.67)
Between patient SD	8.53	–	–	–
Within patient SD	3.94	–	–	–
Intraclass correlation	0.82	–	–	–

Paranoia (GPTS – Part B)				
Green Paranoid Thoughts Scale Part B baseline	0.39	0.27	0.145	(0.13, 0.91)
CBT worry intervention	−10.50	9.23	0.255	(−28.59, 7.60)
Time	4.12	3.08	0.181	(−1.91, 10.14)
Time by CBT worry intervention	−3.27	4.47	0.465	(−12.02, 5.49)
Constant	29.08	15.10	0.054	(−0.52, 58.67)
Between patient SD	18.61	–	–	–
Within patient SD	6.90	–	–	–
Intraclass correlation	0.87	–	–	–

**Table 4:** Association between changes in worry and paranoid thoughts.

	Measure	*n*	Kentall's tau	*p*-value
PSWQ baseline to 1 month change	GPTS – distress subscale, change 0–1 month	19	0.329	0.053
	PSYRATS – Factor 2 distress subscale, change 0–1 month	20	0.229	0.196
	GPTS Part B (persecutory thoughts), change 0–1 month	20	0.390	0.018

PSWQ baseline to 2 month change	GPTS – distress subscale, change 0–2 months	20	0.381	0.021
	PSYRATS – Factor 2 distress subscale, change 0–2 months	20	0.488	0.005
	GPTS Part B (persecutory thoughts), change 0–2 months	20	0.342	0.038
